# Analysis of Available Nutrition Recommendations to Combat COVID-19: A Scoping Review

**DOI:** 10.21315/mjms2021.28.3.3

**Published:** 2021-06-30

**Authors:** Norsyamlina Che Abdul Rahim, Jayvikramjit Singh Manjit Singh, Munawara Pardi, Ahmad Ali Zainuddin, Ruhaya Salleh

**Affiliations:** Institute for Public Health, National Institutes of Health, Ministry of Health Malaysia, Shah Alam, Selangor, Malaysia

**Keywords:** COVID-19, pandemic, nutrition recommendation, nutrition intervention, nutrition prevention

## Abstract

The current COVID-19 pandemic remains severe. There is no doubt that the COVID-19 pandemic is affecting every aspect of our lives. Currently, the spread of inaccurate information or fake news on the internet to the public is causing the community to panic. Thus, this study aims to obtain available information on food and nutrition related to the prevention and treatment of COVID-19 from various sources. A scoping review framework was used to chart the evidence on nutritional recommendations to prevent COVID-19 based on the preferred method in reporting systematic review and meta-analysis extension for scoping reviews (PRISMA-ScR). The articles were categorised into three main groups: i) general dietary recommendations; ii) supplementation with specific micronutrients and iii) their mixtures and supplementation with traditional herbs and miscellaneous foods. A total of 60 articles met the inclusion criteria and were used in the review. This scoping review demonstrates that there is no miracle cure, food or supplement that can cure or prevent COVID-19. Currently, there is no confirmed treatment or vaccine for the disease. Practicing healthy eating habits is the best nutritional recommendation during the pandemic. Hence, this review hopefully will provide evidence-based nutrition recommendations that are available for current COVID-19 treatment. We hope that the authorities can inform the public and media to stop the spread of nutrition pseudoscience in the wake of the COVID-19 pandemic.

## Introduction

The current COVID-19 pandemic remains severe. Currently, the World Health Organization (WHO) reported that the numbers of laboratory-confirmed COVID-19 cases and deaths worldwide were 722,202 and 33,976, respectively (1). It is important to note that these figures are likely to be underestimated given that the data presented exclusively represent laboratory-confirmed diagnoses. At present, there are no specific antiviral drugs or vaccines against COVID-19 infection for potential therapy for humans (2).

Most of the infected patients have developed mild symptoms, such as dry cough, sore throat and fever. Most of the cases have spontaneously resolved (3). However, some cases have developed various fatal complications, including organ failure, septic shock, pulmonary edema, severe pneumonia and acute respiratory distress syndrome (ARDS), that require specialised management in intensive care units (ICUs) (4). Recently, nutritional interventions for COVID-19 have been extensively discussed. A considerable amount of information has been discussed about the possible therapeutic effects of certain properties in food that are able to aid the treatment of COVID-19. Most of the studies and literature that are available are reviews, systematic reviews, observational studies, laboratory diagnosis, case studies, clinical trials and general nutritional guidelines. Currently, there are no definite recommendations available in terms of medical and nutritional therapeutic guidelines for the treatment of COVID-19.

The public is sharing nutrition remedies that claim to cure or prevent this virus without support from evidence-based studies. Malaysians are not left out of this situation of promoting certain dishes, leaves and products that can prevent a person from being infected with COVID-19. Thus, the study aims to obtain available information on food and nutrition related to the prevention and treatment of COVID-19 from various sources. We also analyse and discuss the approaches presented.

## Methods

Scoping reviews are broad by nature and are used to delineate, map the key concepts underpinning a field of research and clarify working definitions and the conceptual boundaries of a topic that encompass a range of interventions and outcome measures (5). This scoping review of empirical research and conceptual literature follows the framework of Arksey and O’Malley (6) and involves the following: i) defining a research question; ii) identifying and selecting relevant studies/ publications; iii) charting resulting data; and iv) interpreting, summarising and reporting the results. The review is published following the preferred reporting items for systematic reviews and meta-analyses extension for scoping reviews (PRISMA-ScR) (7).

### Step 1: Identifying the Research Question

The purpose of this review is to obtain all available food and nutrition information related to the prevention and treatment of COVID-19 from various sources. All literature covering recommendations and claims on nutrients and foods, supplements, herbal remedies and other forms of medical remedies that are suggested for COVID-19 treatment and prevention. We analysed and discussed the approaches presented.

### Step 2: Identifying Relevant Articles and Selecting Articles

A time frame for publications was set to focus on recent developments (1 January 2020– 10 April 2020) related to nutrition myths about COVID-19 from countries with confirmed cases. We performed a search published scientific journals, unpublished work and grey literature noted as below:

- Electronic databases of Medline/PubMed, Science Direct, Web of Sciences and Scopus.
- Relevant research websites, such as the WHO and Google Scholar.
- Bibliographic search of reports, summaries, newsletters and references from selected articles or bulletins from the WHO and Ministry of Health.
- Other sources included information from experts in the relevant agencies and research fields from countries all over the world.

The search was conducted with Medical Subject Headings (MeSH) terms, including novel coronavirus, novel coronavirus 2019, 2019 nCoV, COVID-19, Wuhan coronavirus, SARS-CoV-2, nutrition myth, nutrition therapy, nutrition intervention, nutrition remedies, dishes, leaves, product, food, diet, traditional chinese medicine (TCM), immune system and supplements.

**Figure 1:**
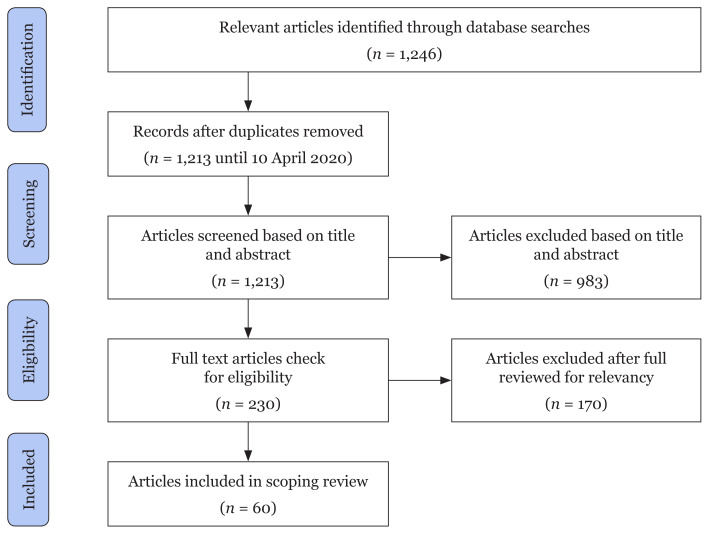
PRISMA-ScR flow diagram for the scoping review process selection of articles

Figure 1 outlines the search strategy. After deduplication, we screened the records to assess eligibility and analyse the full-text manuscripts. The final sample consists of 60 publications (Tables 1 and 2). It is difficult to determine a journal because COVID-19 is a new global pandemic and the role of nutrition in this disease has not been discovered to date. However, many online sources (unpublished) discuss the myths and misconceptions of nutrition.

We used combinations of the following subject headings: COVID-19, nutrition therapy/recommendation/intervention/prevention, nutrition myths and nutrition fallacies. Consequently, the results of our search with MeSH should theoretically include all articles addressing these principles. After screening, the final sample resulting from this MeSH search (*n* = 29) included 31 articles that were already included in the first sample of this scoping review. A vast majority of the articles identified through the MeSH search focused on nutrition recommendations and the relevance of many articles was doubtful regarding the purpose of this scoping review. In every case, we assessed all the fields and subjects addressed by the publications identified through the Google search.

To minimise the risk of omitting relevant sources of evidence, one researcher conducted a search of the grey literature through the internet using different combinations of search terms (8). Grey literature documents are not formally published in academic sources (e.g. peer-reviewed journals) and include information sources such as newspapers, websites, conference proceedings and unpublished research (e.g. theses) (8).

First, a filter was applied to limit the Google search to the region of Malaysia and the Malay and English languages. Next, the first 10 pages of each search’s hits (representing 100 results) were reviewed using the title and two to three lines of text underneath. This number of pages allowed the search to retrieve the most relevant hits while still being a feasible amount to review (8). Potentially relevant records were ‘bookmarked’ in the web browser and later transferred an Excel spreadsheet for further screening. For each search strategy, the search terms, number of results retrieved and screened, and date of the search (1 January 2020–10 April 2020) were recorded. The reference lists of all included sources of evidence were hand-searched by one reviewer to identify additional relevant sources.

### Step 3: Categorising Resulting Data

The articles were categorised as follows: i) general dietary recommendations was the first step in charting the data; ii) supplementation with specific micronutrients or their mixtures (e.g. vitamin C, vitamin D and other vitamins and minerals) and iii) supplementation with traditional herbs of Chinese, Malay or Indian origin and miscellaneous foods that are said to cure and prevent COVID-19.

The next stage involved screening all the publications for a second time to determine whether (or not) these issues were being addressed. We extracted the following data from each record: author(s), year of publication, type of publication, aims of study or subject of the article, study design, outcomes/conclusion, a summary of aspects addressed and keywords.

### Step 4: Collating, Summarising and Reporting the Result

**Figure 2:**
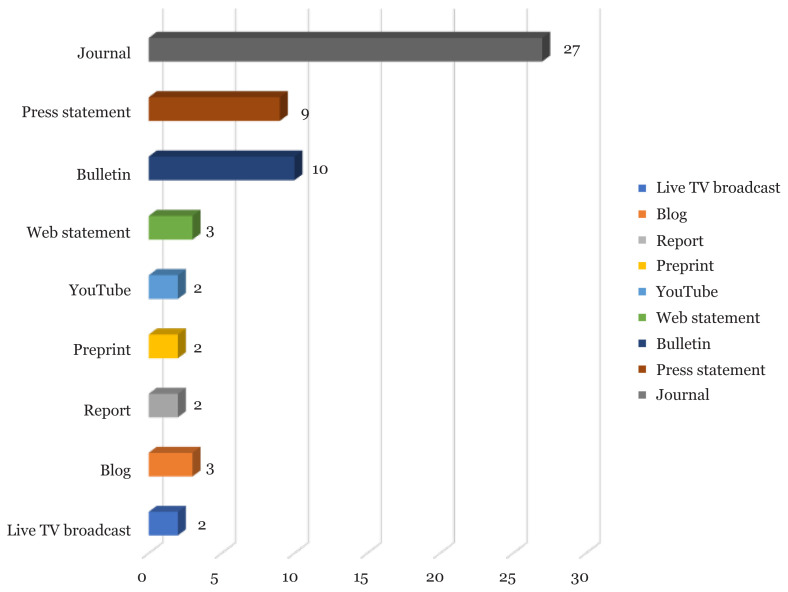
Source of data on nutrition recommendations for COVID-19 (*N* = 60)

Figure 2 summarises the types of data discussed in each nutritional recommendation in COVID-19. Results for each issue are presented, developed and commented in the following sub-sections.

## Results

### Resource Descriptions

The date of publication for included resources ranged from 1 January 2020– 10 April 2020 and 60 resources were published during or available in 2020. For this scoping review, we have searched and analysed available recommendations by other countries inflicted by COVID-19. Of the 60 resources from thirteen countries, 26.5% were from the US (*N* = 16), 25.0% from China (*N* = 15), 10.0% from India (*N* = 6), 6.7% from Malaysia (*N* = 4), 6.7% from Canada (*N* = 4), 6.7% from the UK (*N* = 4), 3.3% from Switzerland (*N* = 2), 3.3% from Iran (*N* = 2), 3.3% from Italy (*N* = 2), 1.7% from Australia (*N* = 1), 1.7% from Finland (*N* = 1), 1.7% from Turkey (*N* = 1), 1.7% from South Africa (*N* = 1) and 1.7% from Indonesia (*N* = 1). Table 1 summarises the potential nutritional recommendations for COVID-19 infection. In total, 60.7% of these studies were identified as review studies, 14.4% observational studies, 10.7% as laboratory diagnoses, 7.1% case studies and 7.1% studies on a clinical trial.

### Evidence-Based on Nutrition Recommendations

In this section, we provide a comprehensive review of the evidence based on nutrition recommendations, including supplementation with specific or mixtures of micronutrient for intervention and prevention of COVID-19 infection that are available in our scoping review.

## General Dietary Recommendations for Intervention and Prevention of COVID-19 Infection

Eleven statements suggested eating healthy and nutritious food (9–19) during a pandemic can help our immune system to function correctly. Generally, people who eat a well-balanced diet tend to be healthier with more reliable immune systems and lower risk of chronic illnesses and infectious diseases (11). Limited access to fresh foods that occurred during quarantine or lockdown may lead to increased total energy intake and the consumption of highly processed foods, which tend to be high in fats, sugar and salt (12). Such changes in eating behaviour related to stress-eating and quarantine situations could harm the immune system, overall physical and mental health, and the well-being of individuals, thus making these individuals more susceptible to viral infection (14).

Three studies generally emphasised consuming plenty of fruits and vegetables to boost immunity to fight viruses or to relieve symptoms of viral infection (12–14). In addition to being a good source of fibre for the gut, consuming plant-based foods helps to improve bowel movement and increases intestinal ‘good’ bacteria, which comprise up to 85.0% of the body’s immune system (12). Meanwhile, a diet that contains high levels of animal foods depletes the body of good bacteria, promotes inflammation and weakens the immune system; thus, these individuals are prone to contagious (viral infection) and non-contagious diseases (cardiovascular diseases, diabetes and cancer) (11). Nutrition Society of Malaysia (NSM) (13) and Australia Associated Press (AAP) (17) suggested increasing fluid intake as part of promoting a balanced gut microbiota and a positive bowel movement to practice healthy eating to fight COVID-19.

## Medical Nutrition Therapy in Patients with COVID-19 Disease

In total, we identified three studies that included standard guidelines on nutrition therapy in the patient with COVID-19 Disease Requiring ICU Care (20–22). The America Society of Parenteral and Enteral Nutrition (ASPEN) produced standardised guidance on nutrition therapy in the patient with COVID-19 Disease Requiring ICU Care, which was released on 30 March 2020. These guidelines emphasise nutrition assessment, nutrition delivery, route of nutrition delivery, nutrition recommendations, formula selection and monitoring for critically ill COVID-19 patients (20).

The Nutrition Therapy Guidelines by Qinggang (21) are also the guidelines that are widely used for supporting treatment for critically ill COVID-19 patients. These guidelines were produced before the ASPEN guidelines. The recommendations are mainly focused on Nutrition Risk in Critically Ill (NUTRIC) assessment on critically ill patients. An interaction among mortality, nutritional intake and the NUTRIC score was noted. Higher NUTRIC scores are associated with increased nutrient intake, thereby reducing mortality, and delaying further complications or symptoms among critically ill COVID-19 patients.

The Enteral Nutrition European Society for Clinical Nutrition and Metabolism (ESPEN) guidelines for COVID-19 proposed 10 practical recommendations to provide concise guidance for the nutritional management of COVID-19. The practical advice is focused on the patients in the ICU setting or older age patients and patients with comorbidities, and these patient populations are independently associated with malnutrition and its negative impact on patient survival (22).

## Supplementation with Specific Micronutrients or Their Mixtures

### Vitamin C

The role of vitamin C in the prevention and treatment of patients with coronavirus has been mentioned and written widely across the world (23–25). A recent study by Taylor (23) recommended a dose of vitamin C of 50 mg–100 mg per kilogram of body weight per day. For severe and critically ill patients, up to 200 mg per kilogram of body weight per day is advised and should be injected intravenously. Supplementation of vitamin C either through diet or tablet was claimed to be a cheaper means of treating disease (24).

Regarding treatment interventions against COVID-19, eight studies (20, 24, 26–31) suggested that high or mega doses of vitamin C have been used intravenously in critical care settings to treat patients with COVID-19. These megadose range from 70 mg to greater than 3000 mg/day. Patients who receive high doses of vitamin C performed better compared to those receiving normal doses of vitamin C (29). One clinical study by Cheng (32) among 50 moderates to severe COVID-19 patients in China claimed that vitamin C and other antioxidants are safe and essentials to mitigate COVID-19 associated with ARDS. Thus, further and well-designed clinical studies should develop standard protocols for the treatment of patients with COVID-19. Cheng (33) also has been actively talking about vitamin C as part of nutritional treatment of COVID-19 in his blog ‘Cheng Integrative Health Centre Blog’ and other media social platforms. Two studies also reported that further studies are needed to determine the mechanics and optimal doses of vitamin C in the treatment of COVID-19 (34–35).

Another clinical trial is a prospective randomised clinical trial of vitamin C infusion for the treatment of severe COVID-19-infected pneumonia patients by ZhiYong (36) of Zhongnan Hospital; the trial started in February 2020 and is recruiting patients until September 2020. These findings will help to determine the efficacy and safety of vitamin C for viral pneumonia. A study by Gupta et al. (37) also claimed that vitamin C plays a role in the prevention of pneumonia, but its effectiveness in COVID-19 requires more research.

In brief, it is understood that none of these papers could provide concrete proof that vitamin C works against COVID-19. Further research is required to determine the exact dosage of vitamin C and the mechanism involved in the treatment of COVID-19 using vitamin C.

### Vitamin D

A total of five studies explored the effectiveness of vitamin D in treating COVID-19 (26, 38–41). Vitamin D is also claimed to be an effective method of treating pneumonia that is especially helpful in cold weather countries, as mentioned in an earlier Grant study (38). Vitamin D can be made in our skin, available in our diet or derived from supplements. Human receive most of their vitamin D from sunshine exposure. Generally, people who are vitamin D deficient have less exposure to sunlight (26). In his study, Andrew (41) suggested maximising the body’s anti-oxidative capacity and natural immunity by consuming vitamin D3. The initial recommended dose is 5,000 IU/day for two weeks followed by a reduction to 2,000 IU daily to prevent and minimise symptoms when a virus attacks the human body.

Four observational studies and expert opinion have expressed the importance of having adequate vitamin D in fighting COVID-19 (38–41). The Former Chief of the US Control Disease Centre (CDC), Dr Tom Frieden reported his opinion that coronavirus infection risk may be reduced by vitamin D in a discussion of the topic of ‘Challenges US faces as COVID-19 pandemic spread’ on the Fox News Channel (39). Grant et al. (38) also claim that a high dosage of vitamin D might be useful to treat patients with COVID-19. In brief, further studies on vitamin D should be performed using randomised controlled trials and large populations to support these claims.

### Vitamins, Micronutrients and Minerals

Five studies suggested specific vitamins, such as vitamins A, B and E, and trace elements to treat COVID-19 (26, 42–45). Vitamin B3 is claimed to have lung-protective effects; therefore, it should be used when COVID-19 patients develop cough (44). These vitamins and trace elements are claimed to play a supporting role in enhancing the immune system to protect against COVID-19 (43).

Eight studies reported that other micronutrients and minerals, such as magnesium, zinc, selenium folic acid with furin and flavonoids, improve viral infection (35, 41, 43, 45–49). Gombart et al. (45) stated that available evidence indicates that supplementation with multiple micronutrients with immune-supporting roles may modulate immune function and reduce the risk of infection. Courtenay (49) also stated that dietary supplements were critical for fighting the disease; however, further studies should be performed using randomised controlled trials and large populations to support this claim.

Luke (46) in his article suggested three important points to maintain vitamin D3, such as the need for an excellent pre-biotic or probiotic, avoiding white sugar and taking zinc and selenium. Andrew (41) from Orthomolecular Medicine News Service also stated that nutritional supplements are essential in fighting the COVID-19 disease. However, a comprehensive hub of evidence-based and fact-checked nutrition information related to the coronavirus pandemic is needed to prove this claim. Meneguzzo et al. (48) also reported that citrus flavonoids aid in the treatment of COVID-19. A study in Iran by Sheybani et al. (47) also found that folic acid with furin should be useful as the safe drug in the prevention or management of COVID-19-associated respiratory disease. Nevertheless, given the lack of randomised controlled clinical trials of any treatment against COVID-19, clinicians are left to utilise therapeutic approaches based on past research.

## Supplementation with Herbal Remedies (Chinese, Malay or Indian)

### Traditional Chinese Medicines

Six studies proposed that TCM should be used to treat COVID-19 patients (50–55). TCM is an ancient system of healing used in China for thousands of years. TCM is a form of health-related practices designed to prevent, treat, and/ or manage illness and/or preserve the mental and physical well-being of individuals (53). A study by Ren et al. (50) stated that TCM is used to treat COVID-19 patients to improve the cure rate, shorten the course of the disease, delay disease progression and reduce the mortality rate. TCM works not only to inhibit the virus but might block infection, regulate the immune response, stop the inflammatory storm and promote the repair of the body (50).

A case study involving four patients in China treated with TCM reported improvements in these patients (51). Chan et al. (52) suggested that given the lack of strong evidence-based regimens, the available data indicate that TCM could be considered as an adjunctive therapeutic option in the management of COVID-19. Chan et al. (52) suggested increasing the contribution and benefits of TCM with more research. Among these studies, TCM showed promising results and we hope ongoing studies on TCM will provide a convincing outcome in the treatment of COVID-19 patients.

### Herbs and Miscellaneous Foods

Table 2 shows the 14 types of herbs and miscellaneous foods that have been claimed to cure or prevent COVID-19 on the internet. Some herbs exhibit several pharmacological activities, such as antibacterial, antiviral, anti-inflammatory, anticancer, cardiovascular and immunomodulatory properties, that play a role in the treatment nausea, dysentery, heartburn, flatulence, diarrhea, loss of appetite, infections, cough and bronchitis. However, no single herb has demonstrated efficacy in the prevention or treatment of COVID-19. This table quotes a comprehensive hub of evidence-based and fact-checked information from previous studies.

## Discussion

This article is the first scoping review that aimed to synthesise a research-based analysis of available nutrition recommendations to combat COVID-19. By having a summary of the literature in this review, health professionals should be able to review current evidence and play their part to promote healthy eating among the population to prevent or reduce the severity of the disease.

### General Nutrition Advice by Various Professional Bodies and Government Agencies to Strengthening Immunity to Combat COVID-19

Following the literature and practice searches, most of the dietitian association societies around the world (13, 16, 18, 22) have emphasised healthy eating habits and general guidelines for food preparation and storage. The American, Canada and Australia Dietetics Association (16, 18) noted that no specific food that can cure and prevent COVID-19 infection. These guidelines also specified good hygiene practices throughout quarantine and lockdown periods. These associations have made no specific claims or unique products related to COVID-19. WHO (65) and UNICEF (10) also encourage general healthy eating guidelines during the lockdown and quarantine period. The Food and Drug Association (FDA) (70) has also made statements that COVID-19 is not foodborne.

Nutrition recommendations from the ASPEN (20) and ESPEN (9) provided convincing evidence-based guidelines for critically ill patients. Malnutrition before and after diagnosis with COVID-19 remains the main focus of medical nutrition therapy when dealing with COVID-19 patients. Therefore, it is important to be aware of nutritional habits during this period and follow a healthy and balanced dietary pattern that contains a high amount of minerals, antioxidants and vitamins. Several studies reported that micronutrients from fruits and vegetables can boost immune function because some of these micronutrients are antioxidants, such as vitamin E, vitamin C and beta-carotene.

This pandemic has emphasised that good nutrition and a healthy life is the key to strengthening immunity. Eating foods that are good sources of immuno-supportive nutrients, and planning times for eating meals, monitoring portions and having positive an attitude could be helpful in addressing the negative health effects of quarantine. Although eating a well-balanced diet can help to ensure the normal functioning of the immune system, no specific nutrient, food or supplement will ‘boost’ it beyond the average level. Therefore, prevention and minimisation of symptoms is more effective than treatments during severe illness phases.

### Importance of the Micronutrient Supplementations, TCM, Specific Herbs and Miscellaneous Foods to Fight against Viral Infection and Specifically COVID-19

Based on our reviews, some convincing claims about vitamin C were made. High dose IVC has been used in China to help improve lung function in people with COVID-19. However, it is essential to note that vitamin C is not yet a standard part of the treatment plan for COVID-19. More research is further warranted in these areas of interest. There is no evidence to support the use of oral vitamin C supplements for COVID-19. Hunt et al. (86) conducted a double-blind trial among elderly patients with severe respiratory infections (bronchitis and bronchopneumonia) and showed that moderate vitamin C supplementation improves clinical progress, especially among severely ill patients on admission. Eventually, vitamin C could help cure bronchopneumonia, lung abscess and purulent bronchitis by improving and restoring normal pulmonary function. This treatment should be given attention and definitely warrants further studies.

Vitamin C may show nonspecific effects on severe viral respiratory tract infections. It also helps to kill viruses and reduces the symptoms of infection (34). Vitamin C affects severe viral respiratory tract infections and significantly lowers the incidence of pneumonia. The possibility that vitamin C affects severe viral respiratory tract infections should be further studied, especially in light of the recent pandemic. Vitamin C acts as an antioxidant, supports immune functions and protects against infection (27). Vitamin C is an essential nutrient found in fruit and vegetables that may help reduce the duration and severity of colds (12).

Vitamin D also enhances immunity, but no specific evidence indicates that these nutritional measures can help protect against or even reduce the effects of COVID-19 infection. Vitamin D modulates the immune system and plays a significant role in protecting and reducing the severity of respiratory lung infections especially among children (predisposes respiratory infections) (41). Vitamin D is also claimed to be an effective method of treating pneumonia and is especially helpful in cold weather countries (38). Epidemiological studies, including several meta-analyses, have shown that people with low vitamin D levels exhibit an increased risk of acute respiratory tract infection and community-acquired pneumonia (41). Vitamin E, vitamin D, and zinc enhance immunity, and more research should be implemented in a clinical setting because there is no evidence that supplements help prevent COVID-19 as stated in most studies. However, there is no specific evidence that these nutritional measures could help protect against or even lessen the effects of COVID-19 infection. Consuming supplements can occasionally be hazardous as the ingredients of the products can cause more harm than good (87). High doses are likely simply excreted through your urine. Exceeding the recommended dosage for specific vitamins and minerals can be detrimental to a person’s health. Drug-nutrient interactions can be fatal if the person consuming it has underlying comorbidities (88). Therefore, we recommend that all vitamins and trace elements should be taken in recommended allowances.

Micronutrients and minerals, such as magnesium, zinc and selenium, have been suggested to improve viral infection (41). Magnesium plays an essential role in vitamin D metabolism, which helps convert vitamin D to its active form (89). Zinc plays a central role in the immune system. Specifically, zinc influences the immune system and alters host resistance to infection (90) and zinc-deficient individuals experience increased susceptibility to a variety of pathogens. Selenium inhibits viral replication (91). Nevertheless, the Ministry of Health Malaysia (92) denies the validity of some health tips, such as consuming certain vitamins, foods, alkaline foods, and hot drinks and sunbathing to defeat the COVID-19 virus. Eating according to the Malaysian Dietary Guidelines as recommended by the Ministry of Health Malaysia ensures the intake of adequate amounts of energy, protein, micronutrients and other food components, which is the key to developing a good immune system.

The WHO (93) has backtracked on using TCM to treat COVID-19. The safety of TCM used in the treatment of emerging coronavirus infections should be carefully evaluated. It is particularly important to avoid toxicity or interference with the efficacy of conventional therapy caused by herb-drug interaction (53). Given the lack of large-scale studies and clinical trials on the effectiveness of TCM, we strongly suggest that more research is needed on the usage of TCM for COVID-19 treatment. Among other recommendations, TCM showed promising results and we hope ongoing studies on TCM will provide convincing data for the treatment COVID-19 patients (50–53).

Drinking hot water can kill the virus is another misunderstanding. Drinking water is essential to maintain the balance of body fluid that optimise body function. Two published articles stated that throughout this COVID-19 pandemic drinking more water has been proposed as part of avoiding the virus infections (19, 62). Japanese doctors treating the COVID-19 cases claimed that regular sips of water can prevent the virus from entering the respiratory system. However, these claims were reported in a false report by the Discover Australia Associated Press (AAP) (17), and the preventative effects were not measured using COVID-19 guidelines by the Japanese Ministry of Health, Labour and Welfare. Nevertheless, hydration is vital to general health and more fluid intake is essential during infection. The novel coronavirus can be killed in water at 56 °C or higher after 30 min. Still, the human body cannot maintain a temperature of 56 °C (81). In their key messages and general guidelines for the nutritional management of COVID-19, Xu et al. (15) recommended fluid intake based on COVID-19 symptoms that are noted by the progression of the infection.

There is also a misconception that several types of foods can cure or treat COVID-19 patients. Among these misconceptions is *Ikan Singgang* dish may have ingredients to fight COVID-19. *Ikan Singgang* might potentially stop COVID-19 because it contains antimicrobial ingredients. Ingredients such as galangal, turmeric, ginger and garlic, which are found in the dish destroy microbes as demonstrated by previous research. Although galangal, turmeric, ginger and garlic are healthy foods that can provide health benefits as part of a balanced diet, there is no evidence from the current outbreak that consumption of these foods has protected people from the new coronavirus (93). With many people sharing unsourced ‘advice’ about miracle supplements and foods that prevent infection, this article produced a comprehensive focal point of evidence-based and fact-checked nutrition information related to the coronavirus pandemic.

The available nutrition recommendations that were listed and searched in all the search engines were thoroughly studied for evidence-based literature support. Unfortunately, none could provide concrete and precise evidence to support nutritional values, properties or cures in the treatment of COVID-19. This information is constantly being updated and the authors are keen to get it into the hands of as many people as possible. It is vital that we stop spreading this misinformation. The best way to protect yourself is simple healthy habits, such as sleeping, exercising, eating well and finding good ways to reduce stress.

## Conclusion

This article produced a comprehensive focal point of evidence based on nutrition recommendations to combat COVID-19. In this scoping review, we highlight the essential facts in this field from our perspectives. There is a considerably amount information on food and nutrition to reduce the risk of COVID-19 and help patients recover. However, the evidence for most of these recommendations should be assessed in detail and the health care professionals need to be cautious when recommending this information to the public. It is highly unlikely that a single food or nutrient is able to boost immunity or to prevent COVID-19 infection. During the COVID-19 pandemic, the best strategy is to practice healthy eating based on the principles of balance, moderation and variety. It is especially important to understand that no supplement, diet, or other lifestyle modification other than practicing social distancing, participating in proper hygiene practices and wearing a mask can protect you from getting the COVID-19 virus.

## Figures and Tables

**Table 1:** Summaries of the included studies on the nutrition recommendations for COVID-19 (*N* = 46)

References	Country	Type of publication	Purpose	Study design	Main results
Muscogiuri et al. (14)	Italy	Journal	To suggest nutritional recommendations during COVID-19 quarantine.	Review study	It was keeping foods that are good sources of immuno-supportive nutrients. Planning times to eat, meals and portions. Having a cut-off time for eating.
Gupta et al. (37)	India	Journal	To review on Vitamin C for lung protection	Review study	Vitamin C supplementation has some role in the prevention of pneumonia and its effect on COVID-19 needs evaluation.
Nutrition Society of Malaysia (13)	Malaysia	Web statement	To recommend healthy eating tips during COVID-19.	Not available	Eat balanced meals. Consume more vegetables and fruits. Guard gut. Adopt healthy cooking practices. Keep physically active even during movement control order (MCO).
Luke (46)	India	Press statement	To boost immunity and prevent COVID-19.	Not available	Take antiviral foods in the diet: – Garlic. – Ginger. – Star anise. – Coconut oil. – Resveratrol. – Vitamin C rich foods. Take antiviral herbs in diet: – Oregano. – Tulsi. – Dried thyme. Maintain vitamin D3: – Need an excellent pre-biotic or probiotic. – Avoid white sugar. – Take mineral (zinc and selenium).
Xu et al. (15)	China	Journal	To summarise and establish an effective treatment strategy centered on “Four-Anti and Two-Balance” for clinical practice.	Laboratory diagnosis	Nutritional support and application of prebiotics or probiotics were suggested to regulate the balance of intestinal microbiota and reduce the risk of secondary infection due to bacterial translocation. The “Four-Anti and Two-Balance” strategy included: – antivirus. – anti-shock. – anti-hypoxemia. – anti-secondary infection. – maintaining of water. – micro ecological balance. – electrolyte balance. – acid-base balance.
WHO (9)	Switzerland	Web statement	To recommend nutrition advice during COVID-19 outbreak.	Not available	Eat fresh and unprocessed foods daily. Drink enough water daily. Eat moderate amounts of fat and oil. Eat less salt and sugar. Avoid eating out.
UNICEF (10)	US	Web statement	To recommend healthy eating tips during coronavirus disease.	Not available	Keep up fruit and vegetable intake. Swap in healthy dried or canned alternatives when fresh produce is not available. Build up a stock of healthy snacks. Limit highly processed foods. Do cooking and eating fun and meaningful part of your family routine.
American Society for Nutrition Member Contributor (18)	US	Bulletin	To recommend general eating guidelines and storage of food.	Not available	Suggested a guideline recommendation for healthy eating and food storage.
Dietitians of Canada (16)	Canada	Bulletin	To recommend general guidelines for food preparation, hygiene, and storage.	Not available	Suggested a guideline for food preparation, hygiene and storage.
Grant et al. (38)	Switzerland	Journal	To review studies on vitamin D in reducing the risk of COVID-19.	Review study	Observational studies and clinical trials reported vitamin D supplementation reduced the risk of influenza. Evidence supporting the role of vitamin D in reducing the risk of COVID-19 includes that the outbreak occurred in winter, a time when 25-hydroxyvitamin D [25(O.H.)D] concentrations are lowest; that the number of cases in the Southern Hemisphere near the end of summer is short. Randomised controlled trials and extensive population studies should be conducted to evaluate these recommendations.
Calder et al. (43)	UK	Journal	Optimal nutritional status well-functioning immune system- to protect against viral infections.	Review study	Supplementation with the micronutrients and Omega-3 fatty acids is a safe, effective, and low-cost strategy to help support immune function. Vitamins (A, B6, B12, C, D, E) and folate, trace elements (zinc, iron, selenium, magnesium), and copper and Omega-3 fatty acids are essential in supporting the immune system.
Gombart et al. (45)	US	Journal	To provide an overview of the known mechanisms of micronutrients.	Review study	Evidence indicates that supplementation with multiple micronutrients with immune-supporting roles may modulate immune function and reduce the risk of infection. Micronutrients with the most robust evidence for immune support are vitamin C, vitamin D and zinc.
Andrew (24)	US	Journal	To maximise the body’s anti-oxidative capacity and natural immunity to prevent and minimise symptoms when a virus attacks the human body.	Review study	Vitamin C intake: 3,000 mg (or more) daily, in divided doses. Vitamin D3: 2,000 International Units (IU) daily. (Start with 5,000 IU/day for two weeks, then reduce to 2,000). Magnesium: 400 mg daily (in citrate, malate, chelate, or chloride form). Zinc: 20 mg daily. Selenium: 100 mcg (micrograms) daily.
Luo et al. (54)	China	Journal	To review on Chinese Medicine (CM) be sued for prevention of COVID-19.	Review study	The main principles of CM use were to “tonify qi” to protect from external pathogens, disperse wind and discharge heat, and resolves dampness. Chinese herbal formula could be an alternative approach to prevent COVID-19 in a high-risk population. The use of CM as preventive measures for COVID-19. Most frequently used in China were: – Radix astragali (Huangqi). – Radix glycyrrhiza (gancao). – Radix saposhnikoviae (Fanfeng). – Rhizome atractylodis Macrocephalae (baizhu). – Lonicerae Japonicae Flos (Jinyinhua). – Fructose forsythia (Lianqiao).
Institute for Functional Medicine (IFM) Medical Education Team (12)	US	Report	To boost immune function and provide symptom relief during illness may help to shorten the duration of illness.	Not available	Brightly coloured vegetables and fruits boost immunity better than most supplements. Eat plenty of fruits and vegetables (aim for ten servings per day). Include fermented vegetables or other probiotic-containing foods.
Restrepo (11)	US	Bulletin	i. To improve immune system plant-based foods. ii. To increase and help the intestinal “good” bacteria, and the overall gut microbiome health.	Not available	Eating organic whole plant foods with the right amounts of leafy greens and fiber-rich foods (legumes, whole grains, beans and vegetables). Avoiding animal products including (poultry, fish, pork, beef and dairy). Adding a lactobacillus probiotic to the everyday routine.
Frieden (39)	US	Bulletin	To review on vitamin D may be reduced by risk COVID-19 infection.	Review study	Vitamin D may reduce the coronavirus infection risk with these suggestions: – Go outside for sunlight. – Eat food naturally rich in vitamin D [e.g., egg yolk and fatty fish (salmon)]. – Take vitamin D supplement.
Australia Associated Press (AAP) FactCheck (17)	Australia	Press statement	To provide facts on COVID-19 drinking water, advise dry on evidence.	Not available	Stay hydrated by drinking water is vital for overall health, but it does not prevent COVID-19 infection.
ZhiYong (36)	China	Journal	To study the clinical efficacy and safety of vitamin C for viral pneumonia through randomised controlled trials.	Clinical trial	Vitamin C infusion can help improve the prognosis of patients with SARI. It is necessary to study the clinical efficacy and safety of vitamin C for the clinical management of SARI through randomised controlled trials during the current epidemic of SARI.
Zhang and Liu (26)	China	Journal	To find alternative methods to control the spread of disease.	Review study	Nutritional interventions (Vitamins A, B, C, D, E, PUFA, Selenium, Zinc, Iron). It was proposed to verify the nutritional status of COVID-19 infected patients before the administration of general treatments. Immunise the current children’s RNA-virus vaccines, including influenza vaccine for uninfected people and health care workers. Convalescent plasma should be given to COVID-19 patients if it is available. Implement all the potential interventions to control the emerging COVID-19 if the infection is uncontrollable. The nutritional status of the host, until recently, has not been considered as a contributing factor to the emergence of viral infectious diseases.
Qinggang (21)	China	Journal	To validation of the “NUTRIC” nutritional risk assessment tool in Chinese ICU patients diagnosed as COVID-19.	Observational study	There was an interaction between mortality, nutrient intake and the Nutrition Risk in Critically ill (NUTRIC) score suggesting that those with higher NUTRIC scores benefited the most from increasing nutritional intake.
British Dietitian Association (22)	UK	Bulletin	To recommend guidelines for nutrition management for critically ill.	Not available	Suggested a guideline for nutrition management for critically ill.
Ren et al. (50)	China	Journal	To report effectiveness on TCM in treating the patient with COVID-19.	Case study	Early intervention of TCM is a crucial way to improve the cure rate, shorten the course of the disease, delay disease progression, and reduce the mortality rate. TCM works to inhibit the virus, but might block the infection, regulate the immune response, cut off the inflammatory storm, and promote the repair of the body. The prevention and control measures of COVID-19 fully reflect the ideology of “preventive treatment of disease”.
Wang et al. (51)	China	Journal	To report on TCM that can be used to inhibit COVID-19.	Case study	Two mild and two severe 2019nCoV pneumonia patients were given combined Chinese and Western medicine treatment, three of whom gained significant improvement in pneumonia associated symptoms. The remaining patient with severe pneumonia has shown signs of development by the cut-off date for data collection.
Chan et al. (52)	China	Journal	To review all currently available guidelines, medicines, cohort studies, case studies to consider TCM as an alternative for COVID-19 treatment.	Review study	Lack of strongly evidence-based regimens, the available data suggest that Chinese Medicine (CM) could be considered as an adjunctive therapeutic option in the management of COVID-19.
Yang et al. (53)	China	Journal	To evaluate the supportive care and nonspecific treatment (TCM) to enhance COVID-19 symptoms.	Laboratory diagnosis	The safety of TCM used in the treatment of emerging coronavirus infections should be carefully evaluated. It is particularly important to avoid toxicity or interfere with the efficacy of conventional therapy caused by herb-drug interaction.
Chang (55)	China	Journal	To provide an alternative in the form of TCM since no vaccine is available to treat COVID-19.	Laboratory diagnosis	Recommended increasing the contribution and benefits of TCM with more research.
Gage (29)	US	Bulletin	To report on vitamin C significantly helps the body fight against COVID-19.	Not available	COVID-19 patients who received 16 times vitamin C did significantly better than those who did not get vitamin C. Vitamin C helps the body fight against inflammatory overreaction the body can get from infection when it has the coronavirus.
Mongelli and Golding (30)	US	Bulletin	To report on vitamin C massive doses in helping coronavirus patients.	Not available	Seriously sick coronavirus patients in New York State’s hospitals are being given massive doses of vitamin C based on promising reports that it is helped people in hard-hit China.
Cheng (32)	China	Journal	To assess the treatment of 50 moderate to severe COVID-19 patients in China.	Clinical trial	High-dose IVC has been successfully used in the treatment of 50 moderate to severe COVID-19 patients in China. Vitamin C and other antioxidants are among currently available agents to mitigate COVID-19 ARDS.
Cheng (33)	China	Blog	To observe the Home Treatment Plan for mild COVID-19 or common colds.	Observational study	Dr Cheng’s Home Treatment Plan for mild COVID-19 or common colds: – Vitamin C, 5,000 mg in cold water, drink at once. – Vitamin C, 1,000 mg – 2,000 mg or more in cold water, by mouth, every waking hour until watery diarrhea. – Vitamin D3, 5,000 IU – 10,000 IU daily. – Zinc 50 mg – 100 mg daily. – Omega-3 fats 3,000 mg – 4,000 mg daily. – Magnesium citrate/glycinate 500 – 1,000 mg daily. – Colostrum, 10,000 mg – 20,000 mg daily. – Hydrogen peroxide 3%, nebulising 10–15 min, 3–5 times daily. Low carbohydrate/ketogenic diet. Well-hydrated. Best under the supervision of a qualified integrative medicine or orthomolecular medicine practitioner.
Cheng (31)	China	Journal	To assess the treatment for mild and moderate types COVID-19.	Review study	Recommendation treatment for mild and moderate types of COVID-19 was heparin anticoagulation and high dose vitamin C.
Cheng (27)	China	Journal	To observe an early and high-dose IVC in helping COVID-19 patients.	Observational study	Early and high-dose IVC is quite helpful in assisting COVID-19 patients.
Basiri (42)	Iran	Journal	To observe on vitamin C, vitamin E, multivitamin and antioxidant drugs as a treatment and prevention for COVID-19.	Observational study	Using some injectable vitamins such as vitamin C and oral vitamin E and multivitamin and highly effective antioxidant drugs such as immune syrup under the supervision of doctors are useful as treatments and prevention of COVID-19 disease.
Editorial Review Board (35)	Canada	Journal	To review on effectiveness integrative medicine, treat COVID-19.	Review study	Integrative Medicine is useful and practical. Supplemental vitamin C, both oral and Intravenous (IV), is an excellent and relatively inexpensive and straightforward treatment for both uninfected individuals at home and critically ill individuals in the hospital. Combined with an overall integrative approach to health management, vitamin C, vitamin D, zinc, and other essential vitamins and minerals can effectively prevent and treat COVID-19. However, the mechanisms and relative benefits of different doses, both oral/liposomal and IV, need further study.
Player et al. (34)	Canada	Journal	Discuss vitamin C that maybe can help to kill viruses and reduces the symptoms of COVID-19 infection.	Review study	Vitamin C has worked against every single virus, including influenzas, pneumonia and even poliomyelitis. Vitamin C supports to boost the immune system. Vitamin C helps to kill viruses and reduces the symptoms of COVID-19 infection.
Hemilä and Chalker (25)	Finland	Journal	To determine vitamin C treat SARS and coronavirus.	Review study	Vitamin C may show nonspecific effects on severe viral respiratory tract infections. The possibility that vitamin C affects severe viral respiratory tract infections would seem to further study, especially considering the recent pandemic.
Taylor (23)	US	Report	Shanghai Medical Association recommends high-dose vitamin C for the treatment of COVID-19.	Not available	The dose vitamin C recommended in the consensus is 50 to 100 mg per kilogram of body weight per day. For severe and critically ill patients, up to 200 mg per kilogram of body weight per day is advised, injected intravenously.
Erol (28)	Turkey	Journal	Discuss on intravenous high-dose vitamin C may be the treatment of choice in the early stages of COVID-19.	Not available	IV high-dose vitamin C treatment has significant benefits in the treatment of sepsis and septic shock.
Sheybani et al. (47)	Iran	Preprint	Folic acid may help to prevent respiratory symptoms associated with COVID-19.	Not available	Folic acid with furin, as the safe drug should be useful in the prevention or management of COVID-19 associated respiratory disease.
Meneguzzo et al. (48)	Italy	Preprint	Citrus flavonoids may contribute to inhibit viral infection and replication.	Not available	Citrus flavonoids help in the treatment of COVID-19. But since there are no randomised controlled clinical trials of any treatment against COVID-19, they are left to utilise therapeutic approaches based on past research.
Robert et al. (20)	US	Journal	To recommend nutrition intervention in ICU settings.	Review study	Nutrition recommendations are evidence-based in ICU settings.
Shi et al. (44)	China	Journal	To report on vitamin B3 for lung protection.	Review study	Vitamin B3 for lung protection should be useful in the prevention or management of COVID-19. Requires future research to determine the effectiveness of vitamin B3 against COVID-19.
Andrew (41)	US	Journal	Suggestion on nutritional treatment for COVID-19 from previous experience treatment during Flu pandemic 1919–1920, Swine Flu 1970’s, Bird Flu, SARS.	Review study	Vitamin C best to build the immune system. Magnesium is very cheap and highly beneficial to treat a viral infection. Vitamin D3 was also effective in treating pneumonia. Zinc is a powerful antioxidant and effective in fighting infections for the body. Selenium is an essential and vital antioxidant. Vitamin B complex and vitamin A, a convenient and economically multivitamin.
Adham (19)	Malaysia	Live TV broadcast	Claimed that drinking warm water could prevent COVID-19 infections.	Not available	Maintaining hydration (drinking water content) with drinking water is vital for overall health; it does not prevent coronavirus infection. COVID-19 has spread to countries with both hot and humid climates, as well as cold and dry. This means that COVID-19 can survive in hot and humid climates.
Courtenay (49)	South Africa	Blog	Claimed that nutritional supplements were essential in fighting the COVID-19 disease.	Not available	Dietary supplements are critical for fighting the disease; however, further studies should be done in randomised controlled trials and large populations to support this claim. They hypothesise vitamins and supplements that could reduce the risk and severity of COVID-19 because of their benefits, which seen in another viral or respiratory disease.

**Table 2:** Summaries of the included studies on the herbs and miscellaneous foods recommendation to prevent and cure COVID-19 infection (*N* = 14)

References	Type of food	Myth and fallacies	Facts (quoted from articles)
Free Malaysia Today Reporter (56)	Turmeric	Turmeric can prevent a person from being infected with COVID-19.	Turmeric has been used against Alzheimer’s disease, arthritis, diabetes, ulcer, cancer, hypertension and high blood cholesterol. It has anti-inflammatory, anti-mutagenic, antibacterial, antifungal, anti-protozoal, antiviral, anti-fibrotic and antivenom properties (57). Turmeric’s effects on health are generally centred upon an orange-yellow coloured, lipophilic polyphenol substance called ‘curcumin,’ which is acquired from the rhizomes of the herb (58).
TimePass Machi (59)	Garlic	There is a claim of a healing effect from COVID-19 infection after eating garlic.	Fresh garlic contains vitamins, minerals, and trace elements that are beneficial for human health. Garlic contains essential oil and has several pharmacological activities such as antibacterial, antiviral, anti-inflammatory, anticancer, cardiovascular, and immunomodulatory properties (60). Garlic has been used to treat colds, hay fever, coughs, asthma, abdominal discomforts, etc. Modern herbalists and doctors have been using garlic oil as an ear drop to heal the pain of ear infection (61). Garlic has some antimicrobial properties and claim of healing from COVID-19 infection; however, no evidence to support the claim (62).
Morimoto (63)	Ginger	Ginger boosting the immune system against COVID-19.	Ginger boosting the immune system against viral. It has antioxidant, antibacterial, anti-inflammatory, antithrombotic, and antimicrobial properties. Ginger is used as a spice and medicine for treating nausea, dysentery, heartburn, flatulence, diarrhea, loss of appetite, infections, cough and bronchitis (60).
Bruno (64)	Elderberry	Black elderberry offers a degree of protection against COVID-19.	The level of antibodies was higher in patients receiving the black elderberry extract versus those receiving the placebo, indicating an enhanced defense response in patients infected with the flu virus during an epidemic in Southern Israel. Black elderberries extract effectively treated and helped relieve symptoms of influenza when taken in doses of 175 mg four times daily (65).
Suraya (66)	Neem leaves	Neem leaves are said to cure COVID-19 patients by drinking boiled water soaked in these leaves. Claims of people being tested negative after drinking neem leave the water.	Neem leaves have been used by Indians and Malays due to their anti-inflammatory properties to cure measles, chickenpox and many other diseases (67). No evidence retrieved on the effectiveness of neem-based products. Overconsumption of neem-based tea or drinks may cause new-onset of G6PD deficiency which can lead to haemolytic anaemia (68).
Chhetri (69)	Tea	Three chemical compounds which are methylxanthine, theobromine, and theophylline that can help cure the COVID-19 disease if a person has an average immune system.	Methylxanthines, theophyllines are used in the treatment of airways obstruction, which is caused by health conditions such as asthma and chronic bronchitis (70).
AFP India (71)	Bitter gourd juice	Taking bitter gourd juice will cure COVID-19 within two hours of its consumption.	Bitter gourd is a green vegetable often used in traditional medicine across Asia. A report in India has recommended that the juice of bitter gourd, a vegetable commonly used in traditional medicine, is an effective treatment for the COVID-19 (72). It was ethical to offer unproven interventions with yet unknown efficacy and adverse effects as potential treatment or prevention, keeping in view no vaccine or antiviral were available.
Luke (46)	Star anise	Taking star anise with warm water and added it to teas like green tea or black tea to the built-up immune system against COVID-19.	Pharmacologically relevant attributes of star anise are its shikimic acid content, which is an ingredient in Tamiflu, a popular medication for the treatment of influenza (73).
Azizah (74)	*Ikan singgang* (Malay dish; fish cooked in clear broth)	*Ikan singgang* dish might potentially stop COVID-19 because it contains microbe fighting ingredients such as galangal, turmeric, ginger and garlic, which can kill microbes.	The galangal, turmeric, ginger, and garlic are a healthy food that can bring health benefits as part of a balanced diet; no evidence to support claims (62).
Yasinta and Rosiana (75)	Alkaline food (e.g., lemon, lime, orange, garlic, mango, pineapple)	Alkaline foods will cure COVID-19 infection. The pH value of the novel Coronavirus ranges between 5.5 and 8.5, and thus, one should consume alkaline food that is above the pH level of the virus to prevent its spread.	The human body’s acid-base balance and stays between 7.35 and 7.45. If it becomes too acid or alkaline, that could be life-threatening, and it generally is an indication of a severe health problem, not the underlying cause (76). Viruses do not have pH values. The relation between alkaline foods and the novel coronavirus is baseless (77).
Langer (78)	Colloidal silver	Colloidal silver is proven to cure COVID-19 disease.	Colloidal silver solutions did not show any antimicrobial effect in vitro on the microorganisms, claims of colloidal silver’s antimicrobial potency are misleading, and there is no place for it as an antiseptic (79).
Turak (80)	Sesame oil	Sesame oil able to fend off the COVID-19 disease.	Sesame oil does not kill the coronavirus. Some chemical disinfectants can ruin the new virus on surfaces, including bleach/chlorine-based disinfectants, those with 75 % ethanol, peracetic acid, and chloroform. But those chemicals, it said, have ‘little or no’ impact on the virus and can even cause harm if they encounter skin or a person’s nose (81).
Chilhavy (82)	Coconut oil	Coconut oil destroying viruses, including COVID-19.	Coconut oil consists mainly of medium-chain saturated fatty acids. The biological properties have been widely explored and investigated due to their antimicrobial potentials. The large concentration of medium-chain fatty acids, including lauric acid and its monoglyceride, monolaurin virgin coconut oil (VCO) effective in their mode of actions against pathogenic microorganisms. VCO contains phytosterols that can provide anti-inflammatory, analgesic, and antipyretic effects. Lauric acid and caprylic acid is also present in it are essential for boosting the immune system against viral (83).
Animals Asia Foundation (84)	Bear bile	Bear bile, as an active ingredient, is being used as a treatment for cases of COVID-19.	Bear bile contains the bile acid ursodeoxycholic acid or UDCA. Synthetic UDCA has been produced and used across the world for decades to treat a variety of medical issues. Bear bile has been used to successfully treat respiratory conditions like pneumonia and similar illnesses to COVID-19 for several years with success. There is no evidence to support the claim (85).
